# Impact of diabetes medications and HbA1c levels on abdominoplasty and panniculectomy outcomes

**DOI:** 10.1016/j.jpra.2025.09.028

**Published:** 2025-10-04

**Authors:** Patrick J. Kennedy, Molly A. Olson, Irina I. Kaptsan, Jonathan Bernard, Ben Ormseth, Jeffrey E. Janis

**Affiliations:** a Department of Plastic and Reconstructive Surgery, College of Medicine, The Ohio State University Wexner Medical Center, Columbus, OH, USA; b Department of Plastic Surgery, The University of Texas Southwestern Medical Center, Dallas, TX, USA; c Independent

**Keywords:** Panniculectomy, Abdominoplasty, Diabetic medications, HbA1c

## Abstract

Obesity is a risk factor for diabetes mellitus (DM), which is associated with impaired wound healing and increased surgical site infections (SSI) due to poor vascularity. Patients who undergo abdominoplasties or panniculectomies often have multiple comorbidities, including DM and obesity. Previous research has demonstrated that adequate perioperative glycemic control is crucial to decreasing the risk of postoperative wound complications; however, the impact of diabetic pharmacology and HbA1c levels on postoperative outcomes in patients who undergo abdominoplasties or panniculectomies has not been studied. We conducted a retrospective cohort study on patients aged 18–85 who underwent abdominoplasty or panniculectomy at our institution from July 2014 to April 2023. Patients were stratified by HbA1c levels into four categories: Normal (<5.7), Prediabetic (5.7–6.4), Diabetic (6.5–6.9), and Diabetic with uncontrolled hyperglycemia (7.0–8.9). Diabetic patients were also categorized based on their diabetic medication use. Postoperative outcomes, including SSI, seroma, hematoma, readmission, and reoperation, were analyzed. Among the 594 patients, 16.3 % had DM, and 6.6 % were on anti-diabetic medications. SSIs occurred in 24.2 % of patients, with no significant differences between groups (*p* = 0.28). This study did not establish a clear relationship between diabetes medication regimens and poor postoperative outcomes; however, it highlights the medical complexity of patients who undergo abdominoplasty or panniculectomy and the perioperative glycemic management. Further research is needed to investigate how to better optimize diabetes management for improved surgical outcomes.

## Introduction

Diabetes mellitus (DM) has been associated with surgical site infections, hospital readmissions, decreased wound healing, poor vascularity, and reduced collagen density, which can lead to increased rates of wound dehiscence.^1^, ^2^, ^3^ Patients with DM are at higher risk of postoperative infections due to decreased fibroblast activity, decreased VEGF expression, increased matrix metalloproteinases and underlying vascular disease.^1^ Obesity is a major risk factor for DM; the MESA study showed that individuals with obesity are nearly three times as likely to develop Type 2 DM as compared to those without.^4^ Abdominoplasty is one of the most common cosmetic procedures in the United States, whereas insurance-based panniculectomies are typically performed for significant functional impairment, skin irritation, hygiene issues, and/or recurrent infection, rather than cosmesis.^5^^,^^6^ Despite differences in indications and technique, both panniculectomies and abdominoplasties can improve a patient’s quality of life regardless of complication rates, and are commonly performed in patients with various levels of obesity, and therefore potentially DM, as well.^3^^,^^7^ Literature in orthopedic, general, vascular and spine surgery has demonstrated the relationship between HbA1c levels, blood glucose levels, and surgical site infection.^8^, ^9^, ^10^, ^11^ However, no studies have examined these associations in plastic and reconstructive surgery, despite known risks of diabetes-related wound healing complications.

**Figure 1:**
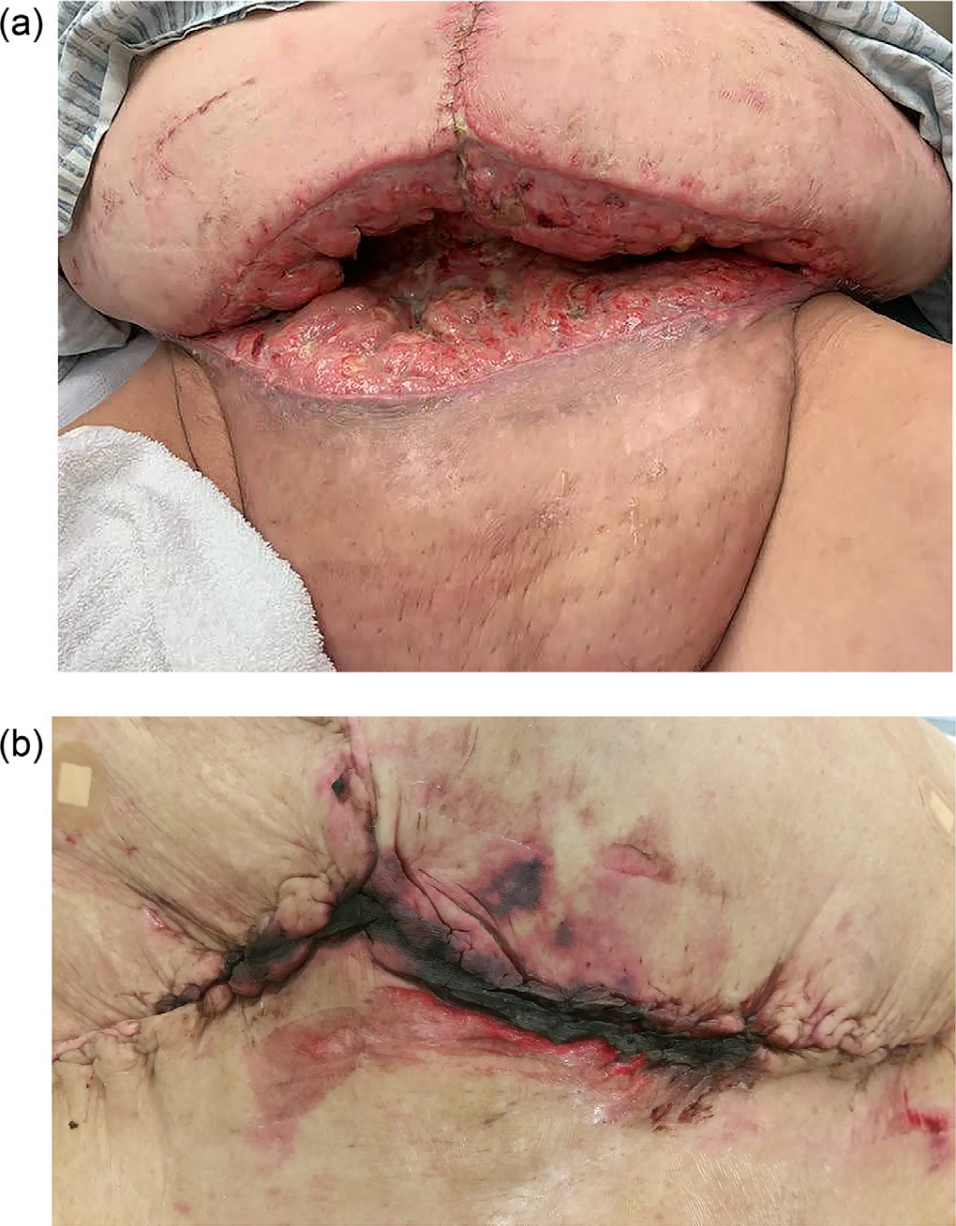

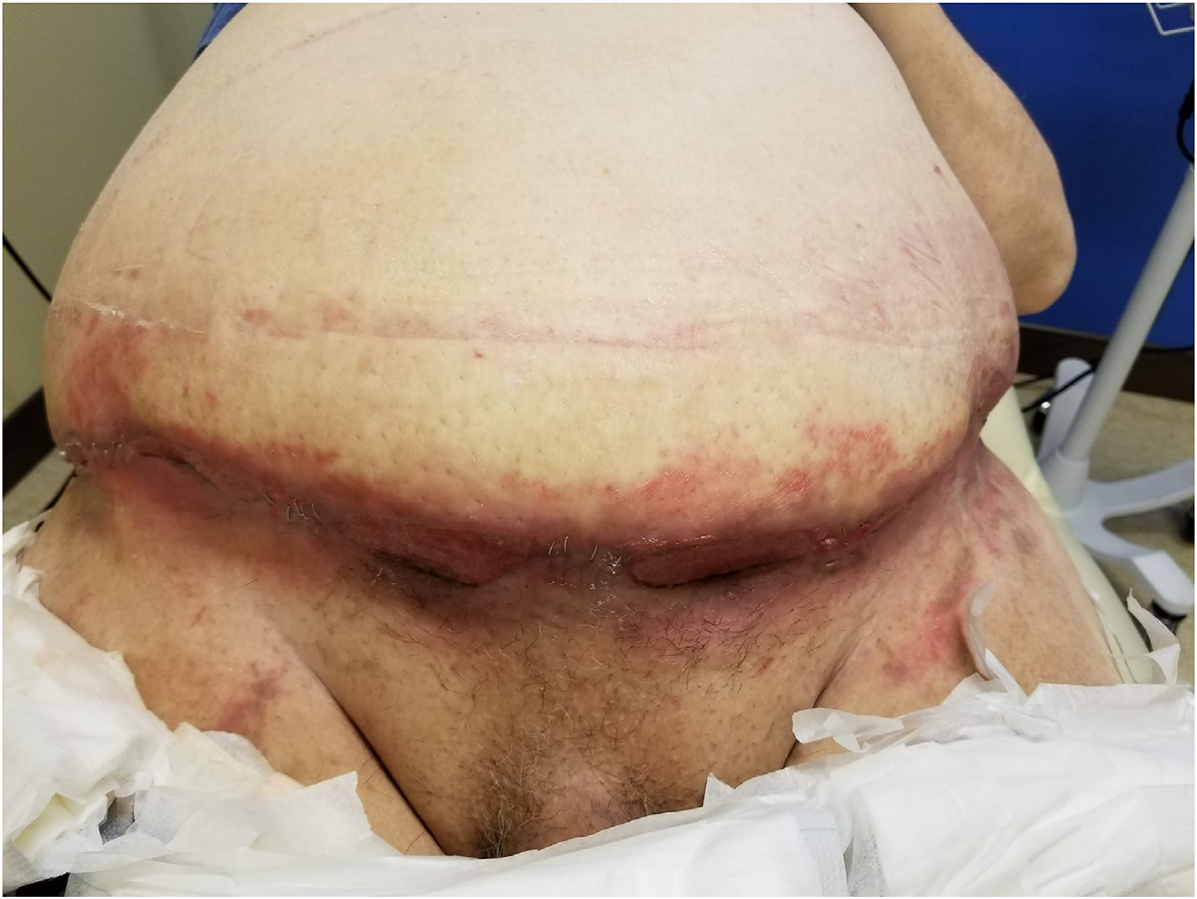
Post-operative complications for panniculectomies and abdominoplasties. a) Panniculectomy surgical site infection and wound dehiscence. b) Incisional necrosis. c) Panniculectomy surgical site infection, wound dehiscence, and peri-incisional ecchymosis.

Abdominoplasty and panniculectomy patients may have multiple comorbidities such as DM, advanced age, obesity, tobacco use, COPD, and hypertension, all of which increase the risk of post-surgical complications as seen in Figure 1.^2^^,^^12^ Exploring strategies that can mitigate the potential consequences of these comorbidities is essential to guiding clinical practice and improving patient outcomes. Basic science research suggests that anti-diabetic agents can help patients maintain stricter glycemic control and therefore improve wound healing.^13^^,^^14^ This can also aid in the reduction of post-surgical site infections if used preoperatively. To date, studies have not examined the impacts of anti-diabetic medications pre- and postoperatively on surgical outcomes in these populations.

This study attempts to address this literature gap by examining the impact of diabetes medications and HbA1c levels on surgical outcomes following abdominoplasty and panniculectomy.

## Patients and methods

### Study design and data collection

We performed a single-site retrospective cohort study using pre-, intra-, and postoperative provider notes, laboratory results, and prescriptions from our institution’s electronic medical records. Patients aged 18–85 who underwent an abdominoplasty or panniculectomy at our institution between July 2014 and April 2023 were included. Patients were excluded who underwent a concomitant intra-abdominal surgery (i.e., hernia repair, hysterectomy, etc.) with a panniculectomy/abdominoplasty, non-diabetics on diabetes medications, patients on more than two medications or those that did not meet treatment group definitions, patients with HbA1c ≥ 9 or missing HbA1c, and patients who died within 6 months of surgery. Relevant variables of their medical and surgical history, as well as medication regimens, were extracted, collected, and managed using REDCap electronic data capture.^15^^,^^16^

HbA1c levels were recorded, if measured within 3 months before or after surgery, while blood glucose levels were recorded only if collected preoperatively to best capture fasting glucose values. The preoperative HbA1c levels were expanded to include values that were taken within 91–180 days of operation date for patients missing HbA1c levels within 90 days. Typically, non-diabetic patients do not have their HbA1c levels collected, these patients were considered to be “Normal” (HbA1c < 5.7) and diabetic. Patients were stratified into four categories: “Normal” (HbA1c < 5.7), “Prediabetic” (HbA1c between 5.7 and 6.4), “Diabetic” (HbA1c between 6.5 and 6.9), and “Diabetic with uncontrolled hyperglycemia” (HbA1c between 7.0 and 8.9).^17^

To account for the variety of treatment options and potential permutations in this population, patients were further stratified by medication regiments. These subcategories, listed in Table 2, include metformin (Glucophage), metformin (Glucophage) with a DPP-4 inhibitor, metformin (Glucophage) with a GLP-1 agonist, metformin (Glucophage) with a sulfonylurea, metformin (Glucophage) with a thiazolidinedione, and metformin (Glucophage) with a SGLT2 inhibitor.

Postoperative data from 6 months was collected to identify complications occurring within the first 30 days and those occurring between 1 and 6 months. We assessed for surgical site infection (SSI), seroma, hematoma, readmission, or reoperation associated with the procedure. This manuscript was checked against the Strengthening the Reporting of Observational Studies in Epidemiology (STROBE) checklist (Supplemental Appendix).

### Statistical analysis

Statistical analyses were conducted to assess the association between diabetic medication regimens and postoperative complications. Baseline characteristics were summarized using mean ± standard deviation and median (interquartile range) for continuous variables and count (%) for categorical variables. Univariate comparisons among the three treatment groups were performed using Kruskal-Wallis for continuous variables and Chi-square test for categorical variables.

Given the small sample size and the potential influence of confounding variables on treatment outcomes, logistic multivariable regression with propensity score covariate adjustment was utilized to balance baseline characteristics across the treatment groups for all outcomes. This method controls for confounding while also performing dimension reduction by summarizing multiple covariates into a single variable, improving model stability and avoiding overfitting. This approach efficiently adjusts for confounders without the need for a large number of parameters, maximizing statistical power and reducing bias in small sample settings.^18^

The propensity score was estimated using a generalized boosted model (GBM). The GBM included the following covariates: age, hypertension, HbA1c (represented as categorical), sex, race, chronic heart failure (CHF), hyperlipidemia, obesity, COPD, peripheral vascular disease, chronic kidney disease (CKD), current cancer treatment, immunocompromised status, chronic liver disease, previous hernia repair, bariatric surgery, or panniculectomy. Covariate balance was assessed using standardized mean differences (SMDs) and the area of common support among treatment groups was evaluated using boxplots of the estimated propensity scores.

All outcomes were modeled using logistic regression. SSI was the only outcome with a sufficient number of events to allow for additional covariate adjustment beyond propensity scores without a high risk of overfitting. The SSI outcome model included HbA1c levels (categorized as normal, prediabetic, diabetic with good control, and diabetic with poor control), CHF, CKD, and obesity, and were selected a-priori based on surgical expertise and expert opinion. Propensity score for metformin (Glucophage) and metformin (Glucophage) + other treatment regimens were included in the regression model with restricted cubic splines with three knots to allow for potential nonlinearity. Seroma, hematoma, reoperation, and readmission outcome models only included the propensity score with restricted cubic splines.

To assess the impact of missing and imputed HbA1c values on our study findings, we performed a tipping point analysis using pattern mixture models. This included analyses to assess the effects of excluding patients with missing HbA1c values and imputing values using the extended 90-day window.

All statistical analyzes were performed using R (version 4.1.1; R Core Team, 2024, Vienna, Austria).

## Results

This study included 594 patients who underwent a panniculectomy or abdominoplasty at our institution during the study timeframe. The mean age of the group was 46.24 years (SD ± 11.72 years). Of these patients, 516 (86.67 %) were female and White (443) and Black or African American (114) were the most patient selected races. Further demographic and clinical characteristics are described in Table 1.

**Table 1:** Baseline demographics.

Variables	N	Overall	No medication	Metformin (Glucophage)	Metformin (Glucophage) + other	*p*
		(*N* = 594)	(*N* = 557)	(*N* = 26)	(*N* = 11)	
Age at time of surgery (years)	594					*p* < 0.01
N		594	557	26	11	
Median (interquartile range)		45.00 (37.00–54.00)	44.00 (37.00–54.00)	56.50 (48.83–61.08)	57.00 (43.67–66.67)	
Range		19.00–82.00	19.00–82.00	34.00–70.00	32.00–74.00	
Mean ± SD		46.24 ± 11.72	45.67 ± 11.56	54.46 ± 9.53	55.64 ± 13.75	
Sex: Female	594	516/594 (86.87)	489/557 (87.79)	18/26 (69.23)	9/11 (81.82)	*p* = 0.02
Race	594					*p* = 0.98
American Indian/Alaska native		3/594 (0.51)	3/557 (0.54)	0/26 (0.00)	0/11 (0.00)	
Asian		3/594 (0.51)	3/557 (0.54)	0/26 (0.00)	0/11 (0.00)	
Native Hawaiian or other Pacific Islander		0/594 (0.00)	0/557 (0.00)	0/26 (0.00)	0/11 (0.00)	
Black or African American		114/594 (19.19)	108/557 (19.39)	4/26 (15.38)	2/11 (18.18)	
White		443/594 (74.58)	412/557 (73.97)	22/26 (84.62)	9/11 (81.82)	
More than one race		13/594 (2.19)	13/557 (2.33)	0/26 (0.00)	0/11 (0.00)	
Unknown / Not reported		18/594 (3.03)	18/557 (3.23)	0/26 (0.00)	0/11 (0.00)	
HbA1c (%), category	594					*p* < 0.01
<5.7, normal		529/594 (89.06)	523/557 (93.90)	4/26 (15.38)	2/11 (18.18)	
5.6–6.4, prediabetes		37/594 (6.23)	28/557 (5.03)	8/26 (30.77)	1/11 (9.09)	
6.5–6.9, diabetic		16/594 (2.69)	1/557 (0.18)	9/26 (34.62)	6/11 (54.55)	
7–8.9, diabetic w/uncontrolled hyperglycemia		12/594 (2.02)	5/557 (0.90)	5/26 (19.23)	2/11 (18.18)	
Peri-operative blood glucose (mg/dL)	203					*p* < 0.01
N		203	169	24	10	
Median (interquartile range)		95.00 (85.00–117.00)	92.00 (84.00–109.33)	136.50 (112.42–156.00)	108.50 (90.33–125.17)	
Range		52.00–257.00	52.00–237.00	77.00–257.00	82.00–184.00	
Mean ± SD		104.61 ± 29.86	99.65 ± 25.15	136.08 ± 40.13	112.80 ± 30.12	
Any comorbidity: Yes	594	472/594 (79.46)	435/557 (78.10)	26/26 (100.00)	11/11 (100.00)	*p* = 0.01
Diabetes: Yes	594	97/594 (16.33)	60/557 (10.77)	26/26 (100.00)	11/11 (100.00)	*p* < 0.01
Congestive heart failure (CHF): Yes	594	18/594 (3.03)	11/557 (1.97)	5/26 (19.23)	2/11 (18.18)	*p* < 0.01
Hypertension: Yes	594	245/594 (41.25)	214/557 (38.42)	23/26 (88.46)	8/11 (72.73)	*p* < 0.01
Hyperlipidemia: Yes	594	170/594 (28.62)	145/557 (26.03)	17/26 (65.38)	8/11 (72.73)	*p* < 0.01
Obesity: Yes	594	392/594 (65.99)	361/557 (64.81)	23/26 (88.46)	8/11 (72.73)	*p* = 0.04
COPD: Yes	594	23/594 (3.87)	19/557 (3.41)	3/26 (11.54)	1/11 (9.09)	*p* = 0.07
Peripheral vascular disease (PVD): Yes	594	9/594 (1.52)	9/557 (1.62)	0/26 (0.00)	0/11 (0.00)	*p* = 0.74
Chronic liver disease: Yes	594	35/594 (5.89)	28/557 (5.03)	4/26 (15.38)	3/11 (27.27)	*p* < 0.01
Chronic kidney disease (CKD): Yes	594	24/594 (4.04)	20/557 (3.59)	3/26 (11.54)	1/11 (9.09)	*p* = 0.09
Cancer diagnosis: Yes	594	15/594 (2.53)	15/557 (2.69)	0/26 (0.00)	0/11 (0.00)	*p* = 0.60
Immunocompromised status: Yes	594	9/594 (1.52)	9/557 (1.62)	0/26 (0.00)	0/11 (0.00)	*p* = 0.74
Previous hernia repair: Yes	594	70/594 (11.78)	64/557 (11.49)	3/26 (11.54)	3/11 (27.27)	*p* = 0.27
Previous bariatric surgery: Yes	594	344/594 (57.91)	329/557 (59.07)	9/26 (34.62)	6/11 (54.55)	*p* = 0.05
Other previous intra-abdominal surgery: Yes	594	500/594 (84.18)	471/557 (84.56)	18/26 (69.23)	11/11 (100.00)	*p* = 0.04
Previous panniculectomy: Yes	594	15/594 (2.53)	15/557 (2.69)	0/26 (0.00)	0/11 (0.00)	*p* = 0.60

A total of 203 patients (34.18 %) had preoperative glucose values, with a mean of 104.61 mg/dL (SD ± 29.86 mg/dL). Among diabetic patients, 37 (6.23 %) had peri-operative HbA1c values of 5.6 %–6.4 %, categorized as “Prediabetic,” 16 (2.69 %) had HbA1c values of 6.5 %–6.9 %, categorized as “Diabetic,” and 12 (2.02 %) had HbA1c values of 7 %–8.9 %, categorized as “Diabetic with uncontrolled hyperglycemia.” There were 529 (89.06 %) patients without a diagnosis of diabetes or diabetic patients with a HbA1c of < 5.7 categorized as “Normal” in this study.

Of the 594 patients, 472 (79.46 %) had at least one diagnosed comorbidity in our electronic medical records, including diabetes mellitus (97, 16.33 %), hypertension (245, 41.25 %), hyperlipidemia (170, 28.62 %), obesity (392, 65.99 %), chronic heart failure (18, 3.03 %), chronic obstructive pulmonary disease (23, 3.87 %), peripheral vascular disease (9, 1.52 %), chronic liver disease (35, 5.89 %), chronic kidney disease (24, 4.04 %), current cancer treatment (15, 2.53 %), or currently immunosuppressed (9, 1.52 %). Additionally, 500 patients (84.18 %) had a history of at least one prior intra-abdominal surgery, with the most common being previous bariatric surgery (344, 57.91 %), hernia repair (70, 11.78 %), or prior panniculectomy/abdominoplasty (15, 2.53 %). Among the diabetic patients, 37 (6.64 %) were prescribed an anti-diabetic regimen. Of these, 26 (4.67 %) patients used metformin (Glucophage) alone, while 11 (1.98 %) took metformin (Glucophage) in combination with additional agents: DPP-4 inhibitors (2, 0.36 %), GLP-1 agonists (3, 0.54 %), sulfonylureas (3, 0.54 %), thiazolidinediones (2, 0.36 %), or SGLT2 inhibitors (1, 0.18 %) (Table 2).

**Table 2:** Medication details.

Variables	N	No medication	Metformin (Glucophage)	Metformin (Glucophage) + other
		(*N* = 557)	(*N* = 26)	(*N* = 11)
Diabetes medications, combination	594			
No medication		557/557 (100.000)	0/26 (0.000)	0/11 (0.000)
Metformin (Glucophage)		0/557 (0.000)	26/26 (100.000)	0/11 (0.000)
Metformin (Glucophage), DPP-4 inhibitor		0/557 (0.000)	0/26 (0.000)	2/11 (18.182)
Metformin (Glucophage), GLP-1 agonist		0/557 (0.000)	0/26 (0.000)	3/11 (27.273)
Metformin (Glucophage), sulfonylurea		0/557 (0.000)	0/26 (0.000)	3/11 (27.273)
Metformin (Glucophage), thiazolidinedione		0/557 (0.000)	0/26 (0.000)	2/11 (18.182)
Metformin (Glucophage), SGLT2 inhibitor		0/557 (0.000)	0/26 (0.000)	1/11 (9.091)

A total of 144 surgical site infections (SSI) were recorded within the 6-month follow up period (24.24 % of patients). Among these, 131 patients taking no anti-diabetic medications had an SSI (23.52 %), nine patients taking metformin (Glucophage) alone (34.63 %), and four patients taking metformin (Glucophage) with another medication (36.36 %) (*p* = 0.28).

Seromas occurred in 23 patients (3.87 %) within the 30-day follow-up period. Of these, 21 patients not taking medications had a seroma (3.77 %), one patient taking metformin (Glucophage) alone (3.85 %), and one patient taking metformin (Glucophage) with another medication (9.09 %) (*p* = 0.66).

Hematomas were recorded in 39 patients (6.57 %) within the 30-day follow-up period. Of these, 36 patients not taking medications had a hematoma (6.46 %), two occurred in patients taking metformin (Glucophage) alone (7.69 %), and one occurred in a patient taking metformin (Glucophage) with another medication (9.09 %) (*p* = 0.91). There were 41 (6.90 %) patients requiring reoperation within 30 days of initial surgery. Of these, 38 patients not taking medications had a reoperation (6.82 %), one patient taking metformin (Glucophage) alone (3.85 %), and two patients taking metformin (Glucophage) with another medication (18.18 %) (*p* = 0.28). A total of 57 patients (9.60 %) required readmission within 30 days of initial surgery. Among these, 53 patients not taking medications required a readmission (9.52 %), two patients taking metformin (Glucophage) alone (7.69 %), and two patients taking metformin (Glucophage) with another medication (18.18 %) (*p* = 0.59). Outcomes data are summarized in Table 3.

**Table 3:** Outcomes.

Variables	N	All	No medication	Metformin (Glucophage)	Metformin (Glucophage) + other	*p*
		(*N* = 594)	(*N* = 557)	(*N* = 26)	(*N* = 11)	
Wound outcomes (SSI): Yes	594	144/594 (24.24)	131/557 (23.52)	9/26 (34.62)	4/11 (36.36)	*p* = 0.28
Seroma within 30 days?: Yes	594	23/594 (3.87)	21/557 (3.77)	1/26 (3.85)	1/11 (9.09)	*p* = 0.66
Hematoma within 30 days?: Yes	594	39/594 (6.57)	36/557 (6.46)	2/26 (7.69)	1/11 (9.09)	*p* = 0.91
Reoperation within 30 days?: Yes	594	41/594 (6.90)	38/557 (6.82)	1/26 (3.85)	2/11 (18.18)	*p* = 0.28
Readmission within 30 days?: Yes	594	57/594 (9.60)	53/557 (9.52)	2/26 (7.69)	2/11 (18.18)	*p* = 0.59

**Figure 2:**
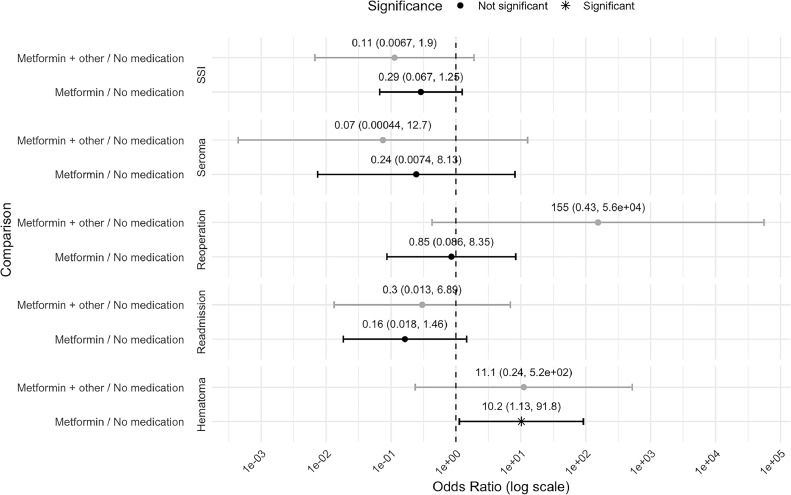
Forest plot depicting the odds ratios (OR) and 95 % confidence intervals (CI) for post-operative complications comparing the use of metformin (Glucophage) with or without another antidiabetic medication vs no medication. Outcomes assessed include surgical site infection (SSI), seroma, reoperation, readmission, and hematoma. Black dots represent not significant results and asterisks indicate statistically significant findings (p < 0.05).

With this sample size, no statistically significant differences were observed in the effect of anti-diabetic medications on the SSI outcome model (*p* = 0.178). The odds ratio estimates for metformin (Glucophage) compared to no medication was 0.29 (95 % CI 0.067, 1.125) and for metformin (Glucophage) and other {with a combination medication} compared to no medication was 0.11 (95 % CI 0.0067, 1.25). Similarly, the overall effect of treatment regimen on seroma, hematoma, reoperation, and readmission outcomes was not statistically significant. However, the data indicated that patients receiving metformin (Glucophage) alone were 10.2 times more likely to develop a postoperative hematoma within 30 days of their procedure compared to patients who did not take medication (*p* = 0.039, 95 % CI 1.13, 91.8). These comparisons are shown in Figure 2.

The tipping point sensitivity analysis demonstrated that our primary findings remained consistent across various imputation scenarios for missing HbA1c values, with no significant changes observed in most outcomes. However, the outcome of hematoma shifted from statistical significance to non-significance when patients with missing HbA1c were included and assumed to have poor glycemic control. A similar shift was observed under a variety of scenarios using imputed HbA1c values for patients with HbA1c measurements within a 90-day window. This indicates a potential tipping point for hematoma under specific assumptions about the missing data.

## Discussion

Through this single center retrospective review, we investigated the association between anti-diabetic medications and the incidence of surgical site infections within the 6-month postoperatively in patients undergoing a panniculectomy or abdominoplasty. Prior research, such as the recent meta-analysis by He et al., has established that as compared to non-diabetic patients, patients with diabetes who undergo abdominoplasty are at a significantly increased risk of major, minor, and overall wound complications.^19^ This study is the first to examine how specific anti-diabetic medications may influence abdominoplasty and panniculectomy outcomes. Although our findings did not demonstrate statistically significant differences among patients’ diabetic medication regimens and postoperative complications, the results highlight the high prevalence of comorbidities in this surgical population.^1^^–^3^,^^12^ Given the complex interplay of diabetes, inflammatory response, anti-diabetic pharmacology, patient comorbidities, and surgical outcomes, these findings underscore the need for further research to determine whether optimizing diabetic pharmacologic regimes preoperatively could mitigate post-surgical complications in this population.^2^^,^^12^

While our study did not identify a significant association between anti-diabetic medication use and surgical site infections within 6-months postoperatively, prior studies suggest that certain anti-diabetic agents may possess anti-microbial properties and promote wound healing. metformin (Glucophage) is an extensively studied medication in this context, with evidence indicating that it reduces infection risk in diabetic patients through mechanisms that remain unclear.^14^^,^^20^^–^23 Additionally, metformin (Glucophage) has been shown to enhance pro-regenerative pathways by decreasing matrix metalloproteinase, upregulating growth factors upregulating angiogenesis growth factors, and promoting epithelization of wounds.^14^^,^^20^, ^21^, ^22^, ^23^ Fewer studies have evaluated the effects of other anti-diabetic agents on wound healing. Xia et al. found that GLP-1 inhibitors had no statistically significant impact of wound healing outcomes, while another study suggested that sulfonylureas may potentially decrease systemic inflammation.^14^^,^^24^

Due to the limited sample size and available data, this study did not compare glycemic control to surgical site infections within the 6-month postoperative period following panniculectomy or abdominoplasty. There is a paucity of research examining the relationship between glycemic control and infectious complications in these procedures.^23^ However, poor glycemic control has been associated with an increased risk of surgical site infections.^25^ Stacey et al. found that diabetic patients have higher rates of resistant pathogen colonization compared to non-diabetic patients.^26^ A 2022 meta-analysis demonstrated that intensive insulin therapy in abdominal and cardiac surgeries significantly reduced the incidence of surgical site infections, while another study suggested that maintaining perioperative blood glucose levels below 150 mg/dl decreases infection risk.^27^^,^^28^ Additional studies report an association between HbA1c levels exceeding 7 %–8 % and increased infection risk, higher hospitalization rates among diabetic patients with poor glycemic control, and an elevated sepsis incidence when comparing HbA1c ≥ 7 % to HbA1c ≥ 11 %.^29^^,^^30^

Sepsis is a rare but serious complication of body contouring procedures, occurring more frequently in functional panniculectomies than in cosmetic abdominoplasties.^31^ Dysregulated immune pathways in diabetes and sepsis may contribute to worsened host response, exacerbated by common type 2 diabetes comorbidities such as obesity and dyslipidemia.^32^ The immunomodulatory effects of insulin and other anti-diabetic medications may play a protective role in diabetic patients with sepsis, warranting further investigation.^33^, ^34^, ^35^, ^36^, ^37^, ^38^, ^39^

This study has several limitations that should be considered when interpreting the findings. First, HbA1c levels were not recorded for patients without a documented diabetes diagnosis. Second, the small sample size and low incidence of the outcomes of interest reduced statistical power, limiting the ability to detect significant differences and generalizability of results. Third, the propensity score model did not consistently achieve acceptable balance thresholds or sufficient overlap among treatment groups, further limiting causal inferences and adjustment for cofounders. These challenges likely stem from a treatment feasibility constraint, where non-diabetic patients would not receive diabetic medications, and patients with more severe disease were more likely to be prescribed these treatments. Future studies with larger sample sizes should include additional adjustments to account for this constraint.

Further investigations should distinguish patient outcomes between undergoing panniculectomies versus abdominoplasties, given the differing medical and cosmetic indications for these procedures. Additionally, incorporating insulin as an anti-diabetic medication in future analysis could provide further insights the severity of diabetes in surgical patients, as insulin is often prescribed when lifestyle modifications and other pharmacologic treatments fail to achieve adequate glycemic control. Finally, the impact of postoperative antibiotic use, including type and duration, should be examined, as this may influence the risk of postoperative infections.

## Conclusion

This retrospective cohort study is the first in plastic and reconstructive surgery to examine the impact of diabetes medications on surgical site infections following panniculectomy and abdominoplasty. While diabetes mellitus is a known risk factor for surgical site infections, this study did not provide sufficient evidence to confirm or refute the association between diabetic medication regimens and infection risk. The relationship remains inconclusive in this study, yet is still important to report. The findings underscore the complexity of diabetes management in surgical patients, where inflammatory response, pharmacologic treatment, and comorbidities interact to influence outcomes. This study highlights the medical complexity for patients who undergo a panniculectomy and abdominoplasty and the importance of preoperative glycemic control to avoid postoperative complications. Prior studies have demonstrated that perioperative glycemic control is a critical factor in the reduction of postoperative surgical site infections and that some anti-diabetic medications improve wound healing. Further research is needed to clarify these relationships and inform perioperative guidelines for diabetes management to optimize surgical outcomes.

## Technical acknowledgments

The authors would like to thank Paul Martinez, MD and Kevin Zhang, MD for their valuable technical assistance on the project.

## Funding

None.

## Ethical approval

This study was approved by our institution’s IRB. The Study Number is 2022H0439.

## Conflict of interest

Dr. Janis receives royalties from Thieme and Springer Publishing. Molly Olson is an independent paid consultant to The Ohio State University. All remaining authors have declared no conflicts of interest No data or information has been presented, wholly or in part at any meetings.

## Appendix Supplementary materials

Image, application 1
