# Supplementary material for: Impact of diabetes medications and HbA1c levels on abdominoplasty and panniculectomy outcomes
Source: JPRAS Open. 2025 Oct 4;46:712–22. doi: 10.1016/j.jpra.2025.09.028 (PMC12661997; doi:10.1016/j.jpra.2025.09.028)
Supplement: Supplementary file 1 [file mmc1.docx]

STROBE Statement—checklist of items that should be included in reports of observational studies

|  | Item No. | Recommendation | Page  No. | Relevant text from manuscript |
| --- | --- | --- | --- | --- |
| **Title and abstract** | 1 | (*a*) Indicate the study’s design with a commonly used term in the title or the abstract | 1 | Impact of Diabetes Medications and HbA1c Levels on Abdominoplasty and Panniculectomy Outcomes |
|  |  | (*b*) Provide in the abstract an informative and balanced summary of what was done and what was found | 2 | Obesity is a risk factor for diabetes mellitus (DM), which is associated with impaired  wound healing and increased surgical site infections (SSI) due to poor vascularity.  Patients who undergo abdominoplasties or panniculectomies often have multiple comorbidities, including DM and obesity. Previous research has demonstrated that  adequate perioperative glycemic control is crucial to decreasing the risk of postoperative wound complications; however, the impact of diabetic pharmacology and HbA1c levels on postoperative outcomes in patients who undergo abdominoplasties or  panniculectomies has not been studied. We conducted a retrospective cohort study on  patients aged 18-85 who underwent abdominoplasty or panniculectomy at our  institution from July 2014 to April 2023. Patients were stratified by HbA1c levels into  four categories: Normal (<5.7), Prediabetic (5.7-6.4), Diabetic (6.5-6.9), and Diabetic  with uncontrolled hyperglycemia (7.0-8.9). Diabetic patients were also categorized based on their diabetic medication use. Postoperative outcomes, including SSI,  seroma, hematoma, readmission, and reoperation, were analyzed. Among the 594 patients, 16.3% had DM, and 6.6% were on anti-diabetic medications. SSIs occurred in 24.2% of patients, with no significant differences between groups (p=0.28). This study did not establish a clear relationship between diabetes medication regimens and poor  postoperative outcomes. However, this study highlights the medical complexity of  patients who undergo abdominoplasty or panniculectomy and the perioperative glycemic management. Further research is needed to investigate how to better optimize diabetes management for improved surgical outcomes. |
| Introduction | | | |  |
| Background/rationale | 2 | Explain the scientific background and rationale for the investigation being reported | 2 | See above |
| Objectives | 3 | State specific objectives, including any prespecified hypotheses | 3 | This study attempts to address this literature gap by examining the impact of diabetes medications and HbA1c levels on surgical outcomes following abdominoplasty and panniculectomy. |
| Methods | | | |  |
| Study design | 4 | Present key elements of study design early in the paper | 4 | We performed a single-site retrospective cohort study using pre-, intra-, and postoperative provider notes, laboratory results, and prescriptions from our institution’s electronic medical records. |
| Setting | 5 | Describe the setting, locations, and relevant dates, including periods of recruitment, exposure, follow-up, and data collection | 4 | Patients aged 18 to 85 who underwent an abdominoplasty or panniculectomy at our institution between July 2014 and April 2023 were included. |
| Participants | 6 | (*a*) *Cohort study*—Give the eligibility criteria, and the sources and methods of selection of participants. Describe methods of follow-up | 4 | Patients aged 18 to 85 who underwent an abdominoplasty or panniculectomy at our institution between July 2014 and April 2023 were included. Patients were excluded who underwent a concomitant intra  abdominal surgery (i.e. hernia repair, hysterectomy, etc.) with a panniculectomy/abdominoplasty, nondiabetics on diabetes medications, patients on more than two medications or those that did not meet treatment group definitions, patients with HbA1c ≥ 9 or missing HbA1c, and patients who died within six months of surgery. |
|  |  | (*b*) *Cohort study*—For matched studies, give matching criteria and number of exposed and unexposed |  | N/a |
| Variables | 7 | Clearly define all outcomes, exposures, predictors, potential confounders, and effect modifiers. Give diagnostic criteria, if applicable | 4 | Postoperative outcomes, including SSI, seroma, hematoma, readmission, and reoperation, were analyzed. Diabetic patients were categorized based on medication use and HbA1c levels. |
| Data sources/ measurement | 8* | For each variable of interest, give sources of data and details of methods of assessment (measurement). Describe comparability of assessment methods if there is more than one group | 4-5 | The study collected data from electronic medical records, including preoperative, intraoperative, and postoperative provider notes, laboratory results, and prescription records. HbA1c levels were recorded if measured within three months before or after surgery, and for patients missing these values, an extended 180-day window was used to capture relevant data. Non-diabetic patients typically did not have HbA1c levels recorded and were assumed to have normal glycemic control unless otherwise documented.  Preoperative glucose levels were only recorded if measured as part of routine clinical care, with 203 out of 594 patients having documented values. Diabetes medication use was determined from electronic prescription records, and patients were categorized into subgroups based on their regimen, including metformin alone or in combination with other agents.  Postoperative outcomes, including surgical site infections (SSI), seroma, hematoma, readmission, and reoperation, were identified through documented clinical diagnoses in provider notes within a six-month postoperative period. Comorbidities and demographic data, such as age, sex, race, hypertension, obesity, chronic heart failure, COPD, chronic kidney disease, and prior surgical history, were also extracted from patient records.  The comparability of assessment methods across different patient groups was ensured through standardized electronic medical record documentation, which provided consistency in data collection. However, variations existed in laboratory testing, as non-diabetic patients were less likely to have HbA1c levels recorded. Additionally, some postoperative complications may have been underreported if they were not clinically significant enough to require documentation in provider notes. |
| Bias | 9 | Describe any efforts to address potential sources of bias | 6 | Selection bias. To minimize bias, propensity score covariate adjustment was used to balance baseline characteristics across treatment groups. |
| Study size | 10 | Explain how the study size was arrived at | 6 | This study included 594 patients who underwent a panniculectomy or abdominoplasty at our institution from July 2014 to April 2023. |

| Quantitative variables | 11 | Explain how quantitative variables were handled in the analyses. If applicable, describe which groupings were chosen and why | 6 | HbA1c levels were categorized into Normal (<5.7), Prediabetic (5.7-6.4), Diabetic (6.5-6.9), and Diabetic with uncontrolled hyperglycemia (7.0-8.9). |
| --- | --- | --- | --- | --- |
| Statistical methods | 12 | (*a*) Describe all statistical methods, including those used to control for confounding | 6 | Statistical analyses included logistic regression models and propensity score covariate adjustments to assess associations between diabetic medication regimens and postoperative complications. A tipping point analysis assessed the impact of missing HbA1c values, demonstrating consistency across various imputation scenarios. |
|  |  | (*b*) Describe any methods used to examine subgroups and interactions | 5-6 | The study used logistic multivariable regression with propensity score covariate adjustment to balance baseline characteristics and assess the association between diabetic medication regimens and postoperative complications. To examine subgroups, patients were stratified based on HbA1c levels into four categories: normal (<5.7%), prediabetic (5.7–6.4%), diabetic (6.5–6.9%), and diabetic with uncontrolled hyperglycemia (7.0–8.9%). Additionally, diabetic patients were further classified based on their medication regimen, which included metformin alone or metformin in combination with DPP-4 inhibitors, GLP-1 agonists, sulfonylureas, thiazolidinediones, or SGLT2 inhibitors.  To explore interactions, the study used a generalized boosted model (GBM) for propensity score estimation, incorporating multiple patient characteristics such as age, hypertension, HbA1c levels, sex, race, chronic heart failure, hyperlipidemia, obesity, COPD, peripheral vascular disease, chronic kidney disease, cancer treatment, immunosuppressed status, chronic liver disease, and prior surgeries. In addition, for the analysis of surgical site infections (SSI), specific covariates—chronic heart failure, chronic kidney disease, obesity, and HbA1c levels—were included in the regression model based on expert clinical input.  To account for missing data, a tipping point sensitivity analysis was performed to assess the potential impact of missing HbA1c values on study findings. This helped determine whether different assumptions about missing data would alter the study’s conclusions. |
|  |  | (*c*) Explain how missing data were addressed | 5 and 8 | The study addressed missing data through a tipping point sensitivity analysis using pattern mixture models to assess the potential impact of missing HbA1c values on the findings. For patients without an HbA1c measurement within 90 days before surgery, an extended 180-day window was used to include values taken up to six months preoperatively. Patients without a recorded HbA1c level were assumed to have normal glycemic control unless otherwise documented.  Additionally, the study explored different imputation scenarios to evaluate whether missing HbA1c values influenced the results. The sensitivity analysis showed that most outcomes remained consistent across various imputation strategies. However, the significance of hematoma risk varied depending on assumptions about missing HbA1c values, suggesting a potential tipping point for this outcome under specific conditions. |
|  |  | (*d*) *Cohort study*—If applicable, explain how loss to follow-up was addressed |  | N/a |
|  |  | (*e*) Describe any sensitivity analyses | 5 and 8 | The study conducted a tipping point sensitivity analysis using pattern mixture models to evaluate the impact of missing HbA1c values on the study's findings. This analysis tested different assumptions about missing data to determine whether changes in these assumptions would significantly alter the results.  To address the absence of HbA1c measurements within 90 days before surgery, an extended 180-day window was used to include values taken up to six months preoperatively. The analysis explored multiple imputation scenarios to assess whether missing HbA1c values influenced postoperative outcomes.  The results of the sensitivity analysis demonstrated that most outcomes remained consistent across different imputation strategies. |
| Results | | | | |
| Participants | 13* | (a) Report numbers of individuals at each stage of study—eg numbers potentially eligible, examined for eligibility, confirmed eligible, included in the study, completing follow-up, and analysed |  | Not Recorded |
|  |  | (b) Give reasons for non-participation at each stage |  | Not Recorded |
|  |  | (c) Consider use of a flow diagram |  | Not Recorded |
| Descriptive data | 14* | (a) Give characteristics of study participants (eg demographic, clinical, social) and information on exposures and potential confounders | 6-7 | The study included 594 patients who underwent abdominoplasty or panniculectomy. Of these, 516 (86.67%) were female. In terms of racial demographics, 3 patients (0.51%) were American Indian or Alaska Native, 3 (0.51%) were Asian, 114 (19.19%) were Black or African American, 443 (74.58%) were White, 13 (2.19%) identified as more than one race, and 18 (3.03%) had no reported race.  Regarding clinical characteristics, 97 patients (16.33%) had diabetes mellitus, while 245 (41.25%) had hypertension, 170 (28.62%) had hyperlipidemia, and 392 (65.99%) were classified as obese. Additional comorbidities included chronic heart failure (18 patients, 3.03%), chronic obstructive pulmonary disease (23 patients, 3.87%), peripheral vascular disease (9 patients, 1.52%), chronic liver disease (35 patients, 5.89%), chronic kidney disease (24 patients, 4.04%), and immunosuppression (9 patients, 1.52%). Furthermore, 15 patients (2.53%) were undergoing active cancer treatment.  A significant proportion of patients had prior surgical history, with 500 (84.18%) having undergone at least one prior intra-abdominal surgery. The most common previous surgeries included bariatric surgery (344 patients, 57.91%), hernia repair (70 patients, 11.78%), and prior panniculectomy or abdominoplasty (15 patients, 2.53%).  Among diabetic patients, 37 (6.64%) were prescribed an anti-diabetic medication. Of these, 26 (4.67%) used metformin alone, while 11 (1.98%) took metformin in combination with other agents. These combinations included DPP-4 inhibitors (2 patients, 0.36%), GLP-1 agonists (3 patients, 0.54%), sulfonylureas (3 patients, 0.54%), thiazolidinediones (2 patients, 0.36%), and SGLT2 inhibitors (1 patient, 0.18%).  The study also identified potential confounders, including age, hypertension, HbA1c levels, sex, race, chronic heart failure, hyperlipidemia, obesity, COPD, peripheral vascular disease, chronic kidney disease, cancer treatment, immunosuppressed status, chronic liver disease, and prior surgeries. These factors were incorporated into the propensity score model to control for baseline differences among treatment groups. |
|  |  | (b) Indicate number of participants with missing data for each variable of interest | 6-7 | HbA1c data were missing for all non-diabetic patients, as these measurements were typically not recorded in this group. Preoperative glucose measurements were missing for 391 out of 594 patients, as only 203 had recorded values. Race information was unavailable for 18 participants. There were no missing data reported for other variables, including patient comorbidities and prior surgical history. |
|  |  | (c) *Cohort study*—Summarise follow-up time (eg, average and total amount) |  | Not Recorded |
| Outcome data | 15* | *Cohort study*—Report numbers of outcome events or summary measures over time | 7-8 | The study included a total of 594 patients who underwent abdominoplasty or panniculectomy. Among them, 144 patients (24.2%) developed surgical site infections (SSI), with 131 cases occurring in non-medicated patients, 9 in those taking metformin alone, and 4 in patients using metformin in combination with other medications.  Seroma formation was observed in 23 patients (3.87%), with 21 cases in non-medicated patients, 1 in the metformin-only group, and 1 in the combination therapy group. Hematomas were recorded in 39 patients (6.57%), with 36 occurring in non-medicated patients, 2 in the metformin-only group, and 1 in the combination group.  Reoperations were required in 41 patients (6.90%) within 30 days of surgery, with 38 cases in non-medicated patients, 1 in the metformin-only group, and 2 in the combination therapy group. Additionally, 57 patients (9.60%) required readmission within 30 days, including 53 non-medicated patients, 2 metformin-only patients, and 2 patients on combination therapy. |
|  |  | *Case-control study—*Report numbers in each exposure category, or summary measures of exposure |  | N/a |
|  |  | *Cross-sectional study—*Report numbers of outcome events or summary measures |  | N/a |
| Main results | 16 | (*a*) Give unadjusted estimates and, if applicable, confounder-adjusted estimates and their precision (eg, 95% confidence interval). Make clear which confounders were adjusted for and why they were included | 6 and 8 | The study provided both unadjusted and confounder-adjusted estimates for the association between diabetic medication use and postoperative complications. In unadjusted analyses, there were no statistically significant differences in surgical site infections (SSI), seroma, hematoma, reoperation, or readmission rates among patients taking no diabetes medications, metformin alone, or metformin with other agents.  After adjusting for potential confounders using logistic regression with propensity score covariate adjustment, the odds ratio (OR) for developing SSI in patients taking metformin alone compared to those not on diabetic medications was 0.288 (95% CI: 0.059–1.138). For patients taking metformin in combination with other medications, the OR for SSI was 0.113 (95% CI: 0.005–1.542). Similarly, for hematoma risk, the adjusted OR for patients on metformin alone was 10.17 (95% CI: 0.989–108.315), suggesting a possible increased risk in this group.  The confounders adjusted for in the regression models included age, hypertension, HbA1c level (categorized as normal, prediabetic, diabetic with good control, and diabetic with poor control), sex, race, chronic heart failure (CHF), hyperlipidemia, obesity, chronic obstructive pulmonary disease (COPD), peripheral vascular disease, chronic kidney disease (CKD), current cancer treatment, immunosuppressed status, chronic liver disease, and prior surgeries (hernia repair, bariatric surgery, or previous panniculectomy/abdominoplasty). These confounders were included based on clinical relevance and expert input, given their known associations with both diabetes and postoperative complications.  The propensity score covariate adjustment was used to improve balance across treatment groups and reduce confounding. The propensity scores were estimated using a generalized boosted model (GBM) and incorporated into the logistic regression models using restricted cubic splines to allow for potential nonlinear relationships. |
|  |  | (*b*) Report category boundaries when continuous variables were categorized | 6 | HbA1c levels were categorized into Normal (<5.7), Prediabetic (5.7-6.4), Diabetic (6.5-6.9), and Diabetic with uncontrolled hyperglycemia (7.0-8.9). |
|  |  | (*c*) If relevant, consider translating estimates of relative risk into absolute risk for a meaningful time period |  | N/a |

| Other analyses | 17 | Report other analyses done—eg analyses of subgroups and interactions, and sensitivity analyses | 9 | A tipping point analysis assessed the impact of missing HbA1c values, demonstrating consistency across various imputation scenarios. |
| --- | --- | --- | --- | --- |
| Discussion | | | | |
| Key results | 18 | Summarise key results with reference to study objectives | 9 | The study did not establish a clear relationship between diabetes medication regimens and postoperative outcomes, but it highlights the complexity of perioperative glycemic management. |
| Limitations | 19 | Discuss limitations of the study, taking into account sources of potential bias or imprecision. Discuss both direction and magnitude of any potential bias | 10 | Limitations include a small sample size, lack of recorded HbA1c levels for non-diabetic patients, and potential confounders affecting treatment groups. |
| Interpretation | 20 | Give a cautious overall interpretation of results considering objectives, limitations, multiplicity of analyses, results from similar studies, and other relevant evidence | 10 | Findings underscore the complexity of diabetes management in surgical patients and the need for further research into optimizing glycemic control for improved surgical outcomes. |
| Generalisability | 21 | Discuss the generalisability (external validity) of the study results | 10 | Results may not be generalizable beyond this single-center study due to its retrospective nature and sample size constraints. |
| Other information | |  | | |
| Funding | 22 | Give the source of funding and the role of the funders for the present study and, if applicable, for the original study on which the present article is based |  | N/a |

*Give information separately for cases and controls in case-control studies and, if applicable, for exposed and unexposed groups in cohort and cross-sectional studies.

**Note:** An Explanation and Elaboration article discusses each checklist item and gives methodological background and published examples of transparent reporting. The STROBE checklist is best used in conjunction with this article (freely available on the Web sites of PLoS Medicine at http://www.plosmedicine.org/, Annals of Internal Medicine at http://www.annals.org/, and Epidemiology at http://www.epidem.com/). Information on the STROBE Initiative is available at www.strobe-statement.org.
